# Spatial and environmental drivers of *Varroa destructor* detection in New South Wales, Australia

**DOI:** 10.1038/s41598-025-28154-8

**Published:** 2025-12-04

**Authors:** Philip P. Mshelbwala, Shannon Mulholland, Tiffany Doyle, Chris Anderson, Shane Hetherington

**Affiliations:** Department of Primary Industries and Regional Development, Orange, NSW Australia

**Keywords:** *Varroa destructor*, Apiculture, Bee health, Honey bee pest, Biosecurity threat, Diseases, Ecology, Ecology, Environmental sciences

## Abstract

*Varroa destructor* is a major global threat to apiculture. Its recent detection in New South Wales (NSW), Australia, triggered an eradicative response followed by a transition to ongoing management. To identify factors influencing the probability of *Varroa* presence across apiaries in NSW, we developed a Bayesian hierarchical logistic regression model, incorporating surveillance data alongside climatic and environmental covariates. Our analysis revealed that detection probability was higher in the eradication emergency zone compared to the general emergency zone and more likely during the summer than winter. Maximum summer temperature was positively associated with *Varroa* presence, while minimum winter humidity was negatively associated with *Varroa* presence. Surveillance methods also influenced detection probabilities, with sticky traps showing higher probabilities than sugar shake methods. Public reports were associated with higher detection probabilities compared to inspections by authorised officers. The probability of detection was lower in areas with registered beekeepers within a 50-km radius compared to areas without registered beekeepers. We observed residual spatial cluster in *Varroa* distribution across the Sydney Basin and extending into the Central Tablelands, suggesting the influence of unmeasured risk factors. These findings highlight the need for targeted surveillance during high-risk seasons and in identified hotspot regions, supported by the wider use of sensitive detection methods and stronger community engagement, to improve early detection and sustainable management of *Varroa* in Australia.

**Supplementary Information:**

The online version contains supplementary material available at 10.1038/s41598-025-28154-8.

## Introduction

Bees and their products are important environmental indicators due to their roles in supporting biodiversity and facilitating pollination of a wide variety of plants^1,2^. As pollinators, bees help ensure the reproduction of many crops and wild plants, which in turn sustain ecosystems^3^. The importance of these pollination-dependent industries cannot be overstated, as they directly contribute to food production, economic stability, and the overall health of the environment. However, despite their vital contributions, bees are facing numerous threats, with one of the most pressing being the *Varroa destructor* mite (hereafter *varroa*) among the most destructive pests affecting honeybee populations worldwide, posing severe threats to bee health, colony survival, and the stability of agricultural ecosystems^4^. *Varroa* attaches itself to honeybees, weakening them by feeding on honeybee fat body tissue^5,6^. This parasitic relationship drains the bees’ strength making them more susceptible to diseases^5^. Moreover, *varroa* is known to be a vector for several harmful viruses, including the Deformed Wing Virus, Acute Bee Paralysis Virus, Kashmir Bee Virus and Sacbrood Virus^7–9^. These viruses, transmitted through the mite’s feeding, exacerbate the already weakened state of the bees and can lead to colony collapse. The presence of these viruses significantly reduces the lifespan and reproductive capabilities of the bees, further destabilising the colony^10^.

Global climate change is having a profound impact on ecosystems, affecting various aspects of biodiversity, including the health and survival of honeybee colonies^11^. These environmental shifts can exacerbate the spread of harmful parasites, such as the ectoparasitic *varroa*.

In June 2022, *varroa* was first detected in sentinel surveillance hives at the Port of Newcastle, New South Wales (NSW), Australia. In response to this detection, a coordinated emergency response was promptly initiated on 23 June 2022, involving both state and local control centres^12,13^. This included the implementation of a 72 h state-wide standstill on the movement of bees, beehives, and bee products, along with plans for the destruction of all wild and managed European honeybee colonies within 10 km of an infested apiary^12^. As part of the emergency response, a zoning framework was established to manage and contain the spread of the pest. This involved the delineation of emergency zones, typically comprising red (eradication), purple (surveillance), and blue (notification) zones, based on the location of infested apiaries^14^. Movement controls were enforced within and between these zones to restrict the spread of varroa, including prohibitions on the movement of hives, equipment, and bee products. Tracing interviews were conducted with affected beekeepers to determine potential pathways of pest introduction and spread, supporting both containment and surveillance efforts^14^. By September 2023, the National Management Group (NMG), the decision making body for national exotic plant pest and disease, shifted its approach to managing *Varroa*, moving away from eradication efforts. The establishment of the pest in Australia could result in annual costs exceeding $70 million due to a reduction in pollination services and the costs of direct Varroa management treatments^12^. Australia was unique in being one of the last major beekeeping nations in the world to remain free of the *Varroa* for decades, while it had already spread throughout other parts of the world since the 1980s. This fact makes the recent detection particularly critical and explains the scale and urgency of the initial eradication response. A preliminary study using limited publicly available data examined the influence of urbanisation, land use, and proximity to incursion sites on *Varroa* detections across NSW^15^. However, this analysis did not account for key factors such as surveillance intensity, climatic variability, or operational constraints. These elements are critical for understanding detection probability and for informing surveillance planning, pest management, and the protection of pollination-dependent agriculture. To address these limitations, our study builds on previous work by incorporating operational surveillance data collected directly by response teams. We used a Bayesian hierarchical logistic regression model to identify the influence of climatic, environmental and epidemiological factors on the probability of *Varroa* detections. Our objective is to enhance understanding of the factors influencing mite presence, thereby informing future biosecurity strategies, improving detection and response capabilities, and supporting the ongoing management of *Varroa* incursions across Australia.

## Methods

### Environmental and epidemiological covariates

We obtained the surveillance data reported via the NSW Department of Primary Industries and Regional Development data collection system, covering the period from September 2022 to July 2025. This data included the latitude and longitude (GPS coordinates) of apiaries. An apiary is defined as a beehive or a group of beehives managed as a single epidemiological unit^16^. The data spanned multiple zones including eradication, surveillance and general zones across NSW^12^. The dataset also provided information on the number of hives at each apiary, the types of surveillance conducted (including Ethanol Wash, Adult Bee Sample, Sugar Shake, and Sticky Trap), laboratory test results (positive or negative), and the months during which the surveillance occurred. At the outset of the response, repeated sampling was conducted on numerous hives across multiple apiaries using different methods to confirm the sensitivity of detection techniques. Once these methods were validated, broad-scale surveillance was rolled out. We also collected information on how surveillance was carried out, whether it was carried out by an authorized officer (response team personnel) or a member of the public. Surveillance by the general public primarily involved alcohol washes, while authorised officers most commonly used sticky mats, alcohol washes, and, in some cases, physical bee or brood samples (targeted physical bee or brood samples from suspect infested apiaries for genetic analysis). Furthermore, the dataset included information on the distance (in kilometers) to the nearest bait station, which served as a monitored lure site used to attract and control wild European honeybees as part of control efforts^12^; the number of registered beekeepers within a 50 km radius of each apiary (from NSW DPIRD registration databases), converted into a binary variable indicating whether there is a beekeeper within the 50 km radius (1) or not (0) and whether the site was used for recreational or commercial beekeeping. Information was also gathered on how beekeepers participated in the ongoing *Varroa* management program, including cases where hives were euthanised as part of the control efforts.

To assess the influence of climatic, environmental and epidemiological factors on the probability of *Varroa* presence across NSW, we supplemented the above data with open source Climatic and environmental data obtained by the Australian Government Bureau of Meteorology and DIVA-GIS^17,18^. We geocoded each apiary’s latitude and longitude coordinates into a spatial object using the World Geodetic System (WGS84) coordinate reference system. We then extracted environmental and climatic data from relevant raster layers using the raster package in R^19^, including monthly temperature (maximum and minimum °C), monthly relative humidity (maximum and minimum), monthly radiation (Watts per square metre), elevation (3 s resolution) and Normalised Difference Vegetation Index (NDVI) and human density. Recent work from Canada has shown that weather variables may be associated with *Varroa* prevalence in a lagged, rather than instantaneous, manner^20^. Given the potential for seasonal variation in weather variables, we calculated seasonal averages for each climatic variable by grouping the data into the four Australian seasons, summer (December–February), autumn (March–May), winter (June–August), and spring (September–November). Seasonal averages were computed using the row Means () function in R^21^. These covariates were selected because climatic, environmental, and epidemiological factors are known to influence *Varroa* mite survival and spread^22,23^. Before statistical analysis, all continuous variables were standardised to unit variance to ensure comparability across regression coefficients. This standardisation was performed using the scale function in R, which involved subtracting the mean and dividing by the standard deviation of each variable. Prior to analysis, variable correlations were assessed using Pearson’s correlation in R (cor() function). Prior to analysis, Pearson’s correlations between variables were calculated in R (cor() function). When two variables were highly correlated (r > 0.75), the variable considered less biologically or epidemiologically relevant was removed to reduce multicollinearity and improve model performance.

### Statistical analysis

#### Model definition

The primary outcome of interest was the presence or absence of *Varroa* mites at individual apiaries across New South Wales (NSW), as determined through surveillance activities conducted during and after the response period (June 2022–July 2025). Apiaries were classified as positive (coded as 1) if *varroa* was detected, or negative (coded as 0) if no mites were found, which was used as our outcome. To investigate factors associated with *Varroa* mite presence, we developed two Bayesian logistic regression models using the brms package (version 2.21.0) in R (version 4.3.3), which interfaces with Stan for full Bayesian inference^24,25^. The models were constructed under the assumption that the observed vector of *varroa* presence across apiary sites in NSW was drawn from a Bernoulli distribution with unknown parameters *p*, representing the probability of *Varroa* presence. We modelled this probability using a logit link function and a linear predictor to estimate the additive effects of environmental, and epidemiological covariates on the probability of *Varroa* presence. The first model assumed that the probability of *Varroa* presence across apiary sites depended solely on epidemiological, and environmental covariates. To account for unmeasured factors influencing *Varroa* detection, the second model extended the first by incorporating a random intercept for postcode, to account for potential spatial clustering of *varroa* within postcodes.

#### Accounting for sparse varroa outcome data and multiple predictors

Given the large number of candidate covariates and our prior assumption that most effects on probability *Varroa* mite presence across apiaries will be near-zero and difficult to estimate with finite outcome *Varroa* data, we guarded against overfitting by applying regularisation to the regression coefficients (β). Regularisation shrinks uninformative coefficients towards zero, producing sparser and more parsimonious models while accounting for interdependencies among predictors. First, we used a modified regularised horseshoe prior, which effectively handles sparsity by shrinking irrelevant coefficients while allowing for larger effects when necessary^26^. This prior is well-suited for models with many predictors, as it adapts to varying levels of importance across them. We also trialled a Student’s t prior for the regression coefficients, which is useful when we expect outliers or extreme values, as it allows for more flexibility in the model and accommodates heavy-tailed distributions^25^. Our examination revealed no difference in model outputs between the two priors, so we proceeded with the horseshoe prior for subsequent analysis.

#### Parameter estimation and model check

The models were run with 4 Markov chains, each with 4000 iterations, including 1000 warm-up iterations. Estimation was performed using Hamiltonian Monte Carlo (HMC) with four independent Markov Chain Monte Carlo (MCMC) chains, each with 2000 iterations, including 1000 warm-up iterations. Convergence was assessed using the potential scale reduction factor (R̂), with values below 1.01 considered indicative of convergence, and further confirmed through visual inspection of trace plots. Model performance was evaluated using posterior predictive checks, which involve comparing simulated data generated from the model’s posterior distribution to the actual observed data. If the simulated data closely match the observed data, it indicates that the model fits well; discrepancies suggest model misspecification. Differences in expected log predictive density (ELPD) were used to compare competing models. Models with higher ELPD values are considered to have better predictive performance. We also computed leave-one-out cross-validation (LOO) to further compare the models. The model with the lower LOO score (indicating better out-of-sample prediction) was considered the best-fitting model. Finally, we then extracted the random intercepts for each postcode to account for spatial clustering. These intercepts were merged with spatial data (postcode, latitude, longitude) and visualized on a scatter plot, with colour representing the magnitude of the intercepts. Areas with higher random effects (indicated by red) suggest a higher likelihood of *Varroa* presence, while areas with lower random effects (blue) indicate a lower likelihood. Covariates were considered to be significantly associated with the outcome if their 95% credible interval (CrI) did not include zero.

## Results

### Surveillance data

A total of 10,118 apiaries with complete data were included in the analysis. Of these, 9.7% (984/10,118) tested positive for *Varroa*. Among the apiaries, 31.2% (3159/10,118) were managed by commercial beekeepers, 39.4% (3,988/10,118) by recreational beekeepers, while the remaining 29.4% (2971/10,118) were unregistered.

Surveillance was predominantly conducted by authorised officers, who performed 85.1% (8617/10,118) of inspections, mostly in eradication zone 52% (5249/10,118), followed by surveillance 25% (2544/10,118) and general zone 23% (2301/10,118). Various surveillance methods were used, including sticky traps 57.3% (5797/10,118), ethanol washes 28.3% (2860/10,118), brood samples (9.2%, 933/10,118), miticide strips 1.9% (195/10,118), and adult bee samples 2.2% (220/10,118).

### Model performance

**Fig. 1:**
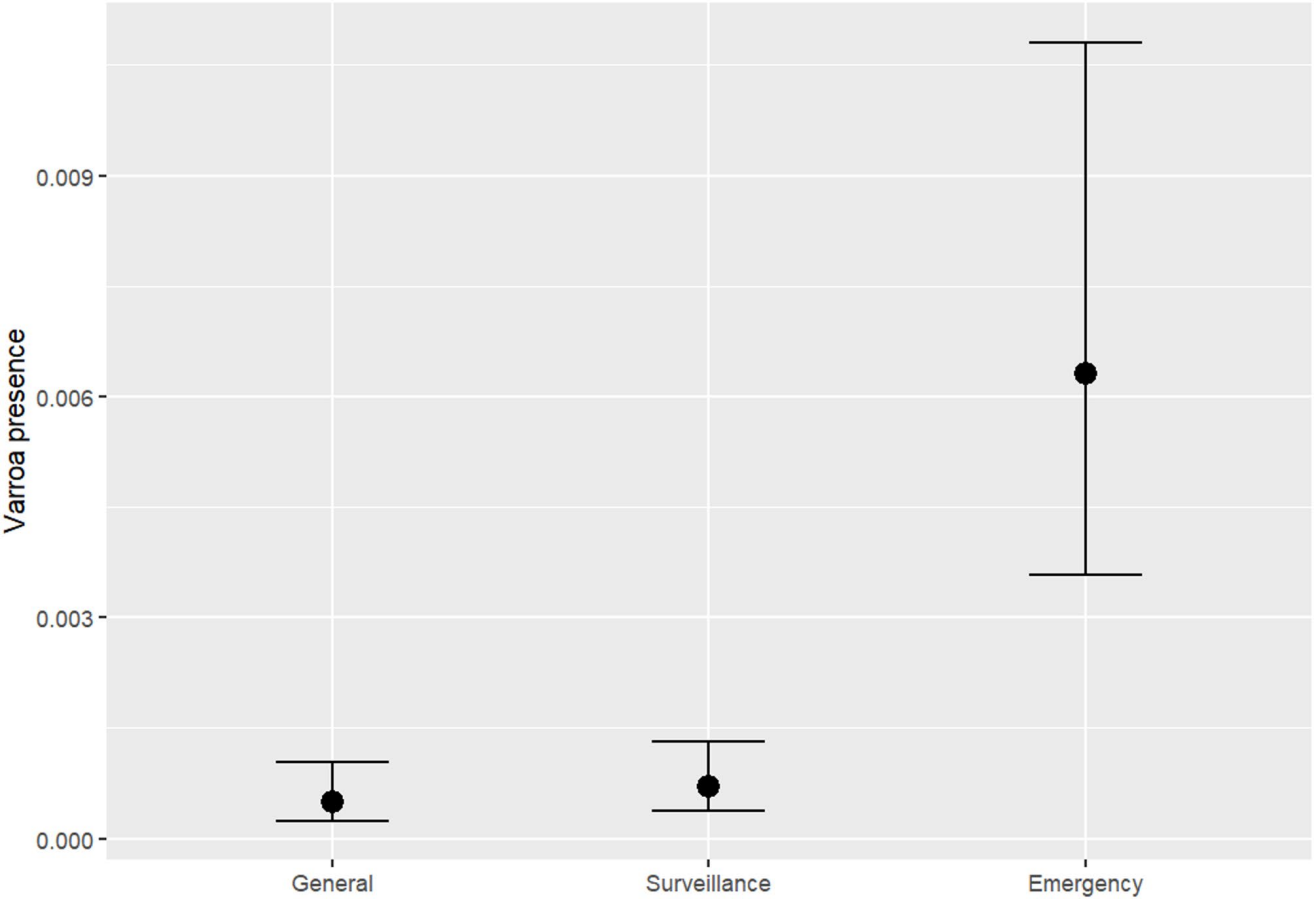
The plot shows the modelled conditional probability of varroa presence across different surveillance types, with the Emergency Zone having the highest probability (0.007), while both the General Zone and Surveillance Zone have the lowest probability (0.001), with all other variables held constant.

Regarding the relative fits of the models to our observed data, Model 2 (with a random intercept) demonstrated good convergence, with all potential scale reduction factors (R̂) below 1.01. Model 2 also showed a higher elpd_waic (estimated log pointwise predictive density) of − 1804.8, suggesting a better fit to the data compared to Model 1 (the fixed-effects model), which had a lower elpd_waic of − 2141.1. Furthermore, Model 2 exhibited a smaller WAIC of 3609.7, indicating better predictive performance and a more favourable balance between model complexity and fit. In contrast, Model 1 had a higher WAIC of 4282.3. Considering the LOO results, Model 2 remains the better model. It achieved an elpd_loo of − 1808.3, higher than the elpd_loo of − 2141.2 for Model 1, indicating superior predictive fit. Additionally, Model 2’s LOOIC (Leave-One-Out Information Criterion) of 3616.6 is lower than that for Model 1 (4282.3), further supporting its better performance. Moreover, Model 2 had a higher p_loo of 142.7, compared to Model 1’s 36.1, which suggests that while Model 2 is more complex, it still provides more accurate predictions. Both models showed good Pareto k diagnostics, indicating that neither model is unduly influenced by problematic data points. Consequently, Model 2 was selected for inference and subsequent analyses, based on its superior predictive accuracy and reliable convergence. Supplementary files include trace plots and the results of the posterior predictive check, both confirming the strong convergence of Model 2 (Fig. 1).

### Factors associated with the probability of the presence or absence of *Varroa destructor* mites

**Fig. 2:**
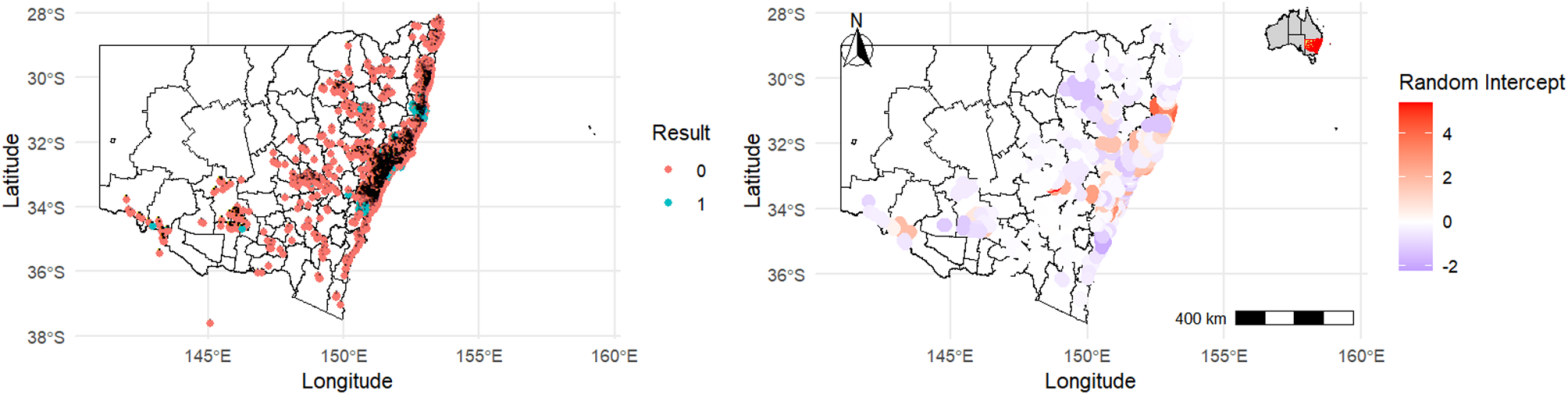
The left panel shows the observed binary outcomes from the New South Wales surveillance data, indicating both negative and positive *Varroa* detections, showing both positive (turquoise blue) and negative (peach) Varroa detections. The right panel displays the estimated random effect, which captures residual spatial autocorrelation in *Varroa* detections. Areas with higher random effect values (e.g., the Sydney Basin extending west into the Central Tablelands) correspond to regions with a higher probability of *Varroa* detections. An inset map in the top right corner highlights New South Wales’ location within Australia. The map was generated using a shapefile from DIVA-GIS, with spatial analysis and visualization conducted in R using the ggplot2 package.

The posterior distribution of model estimates indicated that the probability of *Varroa* presence across infested apiaries was significantly associated with several key factors. Reports by the public showed a higher likelihood of detecting *Varroa* compared to authorised officers (Posterior mean = 1.46, 95% CrI = 1.15–1.77). Commercial beekeepers had a lower probability of *Varroa* presence compared to unregistered beekeepers (Posterior mean = 0.52, 95% CrI = 0.25–0.79). The probability of detection was higher in eradication zones (Posterior mean = 1.94, 95% CrI = 1.34–2.52), with surveillance methods like ethanol wash (Posterior mean = 2.04, 95% CrI = 1.65–2.43) and adult bee samples (Posterior mean = 2.71, 95% CrI = 2.19–3.22) also showing significant associations. The presence of registered beekeepers within 50 km significantly reduced *Varroa* detection (Posterior mean =  − 1.24, 95% CrI =  − 1.84 to − 0.63). Seasonal effects were strong, with summer (Posterior mean = 2.61, 95% CrI = 2.29–2.94), autumn (Posterior mean = 2.31, 95% CrI = 1.94–2.94), and spring (Posterior mean = 2.17, 95% CrI = 1.86–2.49) showing significantly higher probabilities of *Varroa* presence compared to winter. Maximum summer temperature (Posterior mean = 0.43, 95% CrI = 0.26–0.61) was positively associated with *Varroa* presence, while minimum winter humidity (Posterior mean =  − 0.17, 95% CrI =  − 0.28 to − 0.06) was negatively associated, suggesting lower winter humidity reduces *Varroa* detection (Table 1). After accounting for environmental and epidemiological covariates, the estimated spatial random effect of postcode revealed heterogeneity, with posterior mean values on the logit scale ranging from − 2 to 4. As shown in Fig. 2, areas with higher spatial mean values (e.g., the Sydney Basin and west into the Central Tablelands) correspond to regions with an increased risk of *varroa* presence.

**Table 1:** Posterior mean estimates and 95% credible intervals for the effects on the logit (probability) of Varroa presence from a hierarchical Bayesian logistic regression model, including odds ratios.

Variable	Posterior mean (95% CrI)	Odds ratio (95% CrI)
Who performed surveillance		
Authorised officer	Reference	
Public	1.46 (1.15 to 1.77)	4.31 (3.16–5.87)
Beekeeper type		
Unregistered	Reference	
Commercial	0.52 (0.25 to 0.79)	1.68 (1.28–2.20
Recreational	0.03 (− 0.14 to 0.23)	1.03 (0.87–1.26)
Euthanised		
No	Reference	
Yes	− 0.37 (− 7.41 to 6.45)	0.69 (0.0006–632.46)
Zones		
General	Reference	
Surveillance	− 0.07 (− 0.66 to 0.44)	0.93 (0.52–1.55)
Eradication	1.94 (1.34 to 2.52)	6.96 (3.82–12.42)
Surveillance type		
Sugar shake	Reference	
Ethanol wash	2.04 (1.65 to 2.43)	7.69 (5.21–11.39)
Sticky trap	1.17 (0.70 to 1.65)	3.22 (2.01–5.21)
Adult bee sample	2.71 (2.19 to 3.22)	15.03 (8.94–25.05)
Kilometre to bait station	0.22 (0.03 to 0.41)	1.25 (1.03–1.51)
Normalised difference vegetation index	0.01 (− 0.07 to 0.08)	1.01 (0.93–1.08)
Registered beekeepers within 50 kms		
No	Reference	
Yes	− 1.24 (− 1.84, − 0.63)	0.29 (0.16–0.53)
Season		
Winter	Reference	
Summer	2.61 (2.29 to 2.94)	13.60 (9.86–18.97)
Autumn	2.31 (1.94 to 2.94)	10.08 (6.96–18.97)
Spring	2.17 (1.86 to 2.49)	8.76 (6.42–12.07)
Temperature		
Maximum summer temperature	0.43 (0.26 to 0.61)	1.54 (1.30–1.84)
Maximum autumn temperature	− 0.78 (− 1.75, − 0.01)	0.46 (0.17–0.99)
Maximum winter temperature	0.48 (− 0.18 to 1.47)	1.62 (0.84–4.34)
Maximum spring temperature	0.37 (− 0.26 to 1.21)	1.45 (0.77–3.35)
Minimum summer temperature	− 1.06 (− 1.59, − 0.54)	0.35 (0.20–0.58)
Minimum spring temperature	0.22 (− 0.28 to 0.91)	1.25 (0.76–2.48)
Humidity		
Minimum autumn humidity	− 0.05 (− 0.18 to 0.07)	0.95 (0.84–1.07)
Minimum winter humidity	− 0.17 (− 0.28, − 0.06)	0.84 (0.76–0.94)
Minimum spring humidity	− 0.06 (− 0.17 to 0.04)	0.94 (0.85–1.04)
Maximum summer humidity	Removed	
Maximum autumn humidity	− 0.03 (− 0.22 to 0.13)	0.97 (0.80–1.14)
Maximum winter humidity	− 0.13 (− 0.28 to 0.01)	0.88 (0.76–1.01)
Radiation		
Summer radiation	− 0.19 (− 0.31, − 0.06)	0.83 (0.73–0.94)
Autumn radiation	− 0.11 (− 0.33 to 0.07)	0.90 (0.72–1.07)
Winter radiation	0.52 (0.16 to 0.89)	1.68 (1.17–2.44)
Spring radiation	− 0.03 (− 0.17 to 0.09)	− 0.03 (− 0.17 to 0.09)

Crl, Credible interval.

## Discussion

This study provides important insights into the spatial and operational factors influencing the detection of *varroa* based on data collected during the emergency response phase. Our findings demonstrate that spatial modelling can significantly enhance the understanding of *varroa* detection, particularly when traditional fixed-effect models are insufficient.

Our analysis demonstrated that incorporating a spatial random effect significantly improved our model performance compared to a fixed effects only specification. This suggests that including spatial structure improved model fit in the distribution of *Varroa* detections across NSW. The spatial random model likely captures unmeasured spatial heterogeneity arising from geographical clustering, environmental gradients, localised beekeeping practices and spatial distribution of floral resources^27,28^. These findings are consistent with previous investigations, which account for spatial autocorrelation, improved model accuracy and inference^29^.

Our model result also suggests that higher spatial mean values in the Sydney Basin and extending west into the Central Tablelands and mid-north coast correspond to regions with increased risk of *Varroa* presence. This pattern indicates the influence of unmeasured, spatially structured factors driving the distribution of *Varroa* mites. These high-risk areas are geographically close to the outbreak epicentre in Newcastle and the Central Coast, where the mites are actively spreading south into the Sydney Basin and west into the Central Tablelands, aligning with observed trends of spread in NSW. The mid-north coast’s relatively warm and stable climate supports year-round brood production and sustained colony activity, which may support the viability and detectability of *Varroa* populations in that region. Moreover, these regions coincide with areas of high beekeeper activity and overlap major transportation corridors, which may facilitate human-mediated mite spread. There is also a risk of northward spread from the Kempsey cluster due to the population size and availability of suitable habitat along the coast. Surveillance and management efforts should be intensified in these high-risk areas, with targeted efforts along transport corridors and strong engagement with local beekeepers to prevent further spread.

Our analysis found associations between climatic variables and *Varroa* mite detections, which align with findings from earlier studies^23,29,30^. For instance, we observed a positive correlation between maximum summer temperature and the presence of *Varroa* mites, consistent with previous reports^31^. Conversely, minimum winter humidity was negatively associated with *Varroa* presence, further supporting earlier findings. These results suggest that climatic conditions, particularly temperature and humidity, may play a significant role in regulating mite populations, due to the influence on bee reproductive biology. This finding might be due to the impact of temperature on mite reproduction rates, where warmer conditions accelerate their development, while low winter humidity could reduce mite survival, as it affects both mite and bee physiology. Similarly, our model shows a higher probability of detecting *Varroa* during summer compared to winter. This finding underscores the importance of aligning surveillance efforts with seasonal patterns to improve detection accuracy. This finding is consistent with previous studies and is likely driven by increased bee activity and higher mite reproduction rates in warmer conditions^5^. This seasonal trend is likely driven by increased honeybee activity and higher *Varroa* reproduction rates in warmer conditions, which result in larger and more detectable mite populations. This effect is further modulated by regional climate; in areas with warmer and more uniform climates, such as the mid-north coast, with year-round brood production and sustained colony activity, *Varroa* populations may be maintained at detectable levels throughout the year. In contrast, regions with colder, more distinct winters, such as the Central Tablelands, might experience reduced brood availability and bee activity during winter, which can temporarily suppress mite reproduction and make infestations harder to detect. As a result, surveillance conducted in colder months, especially in these cooler regions, may underestimate infestation levels, potentially allowing *Varroa* populations to persist undetected and expand during the following spring and summer. These findings suggest that surveillance efforts should be concentrated during the warmer months, particularly in cooler inland regions, to maximise detection efficiency and improve the chances of early intervention. Strategically timing monitoring activities can enhance the effectiveness of integrated pest management (IPM) strategies, reduce colony losses, and support more sustainable beekeeping practices. Relying on winter inspections may lead to underestimations of infestation levels, potentially allowing Varroa populations to expand unchecked. Strategically timing monitoring can enhance the efficacy of integrated pest management (IPM) strategies, reduce colony losses, and support more sustainable beekeeping practices^32^.

Our result also indicates that the probability of *Varroa* detection was higher when surveillance was conducted by members of the public compared to response team personnel. This finding suggests that public involvement is a valuable and effective source of surveillance. While response teams may have used more sensitive diagnostic methods, the broader reach and scale of public participation likely contributed to a higher overall detection rate. Moreover, providing incentives for reporting, rather than penalties, may have encouraged greater public engagement, following the transition to management in late 2023. This highlights the importance of investing in community-based surveillance as a core component of biosecurity strategy^33^. Public reporting systems should be supported through user-friendly digital platforms, clear communication channels, and targeted education campaigns to improve detection accuracy and timeliness. Strengthening public engagement will foster a culture of shared responsibility and trust between government agencies and stakeholders.

Furthermore, our model result indicates a lower probability of *Varroa* mite detection in areas with registered beekeepers within a 50-km radius than in areas without them. This may be attributed to the potential for more frequent and effective mite management, such as regular hive inspections and targeted treatments, which could reduce the likelihood of detectable infestations. However, as our model did not account for specific management practices, this relationship remains speculative and warrants further investigation. *Varroa* detection rates were lower among commercial beekeepers compared to unregistered beekeepers, likely due to differences in apiary size, location within eradication zones, and exposure to biosecurity trainings^34^.

Our model result demonstrates a lower probability of *Varroa* detection using the sugar shake method compared to Sticky trap, adult bee sampling and ethanol wash. This contrasts with previous studies suggesting that sugar shake is approximately 90% as effective as alcohol wash in recovering mites^35^. The discrepancy may be due to methodological variation, including differences in shaking intensity, duration, sugar type, or the number of bees sampled, all of which can influence mite recovery^36^.

A few limitations should be considered when interpreting these findings. First, the data were collected primarily for operational response purposes, not hypothesis-driven research, which could introduce biases. Surveillance efforts were concentrated in regions with known *Varroa* detections, potentially limiting the representativeness of the dataset and skewing the analysis of broader spatial patterns or environmental associations. Although repeated sampling of multiple hives across several apiaries using different methods was conducted to confirm detection sensitivity, variations in colony health, environmental conditions, and management may have confounded the results, limiting direct comparison of test performance. Spatial autocorrelation suggests that the model did not account for key risk factors, such as informal hive movements, landscape connectivity (e.g., floral resources, wind corridors), and socio-behavioural variables (e.g., beekeeping practices). Lastly, the binary nature of the detection outcome limits the ability to capture the full dynamics of mite infestations, and quantitative data on mite loads would provide more nuanced insights.

## Conclusion

This study highlights the role of spatial and environmental factors in detecting *Varroa* mites in NSW, showing how spatial modelling can improve detection accuracy. Surveillance should focus on high-risk areas like the Sydney Basin, Central Tablelands, and mid-north coast, where environmental conditions, beekeeping activity, and climate influence mite distribution. Seasonal trends, particularly during warmer months, should guide monitoring strategies for better detection and timely intervention. Public involvement in surveillance was also found to increase detection rates, emphasising the value of community engagement in biosecurity efforts. While the findings provide valuable insights, further research is needed to address data limitations and explore additional risk factors, such as informal hive movements and management practices. Effective Varroa management will depend on strategic monitoring, targeted interventions, and stronger public participation.

## Supplementary Information

Below is the link to the electronic supplementary material.

Supplementary Material 1

## Data Availability

The data used in this study contain sensitive information and are therefore not publicly available. However, the raster datasets for climatic and environmental covariates were sourced from publicly accessible repositories. The source for these datasets can be accessed through the following links (Data—DIVA-GIS) and (http://www.bom.gov.au/climate/austmaps/about-agcd-maps.shtml).
