# Supplementary material for: Spatial and environmental drivers of Varroa destructor detection in New South Wales, Australia
Source: Sci Rep. 2025 Dec 4;15:44438. doi: 10.1038/s41598-025-28154-8 (PMC12738786; doi:10.1038/s41598-025-28154-8)
Supplement: Supplementary file 1 — Supplementary Material 1 [file 41598_2025_28154_MOESM1_ESM.docx]

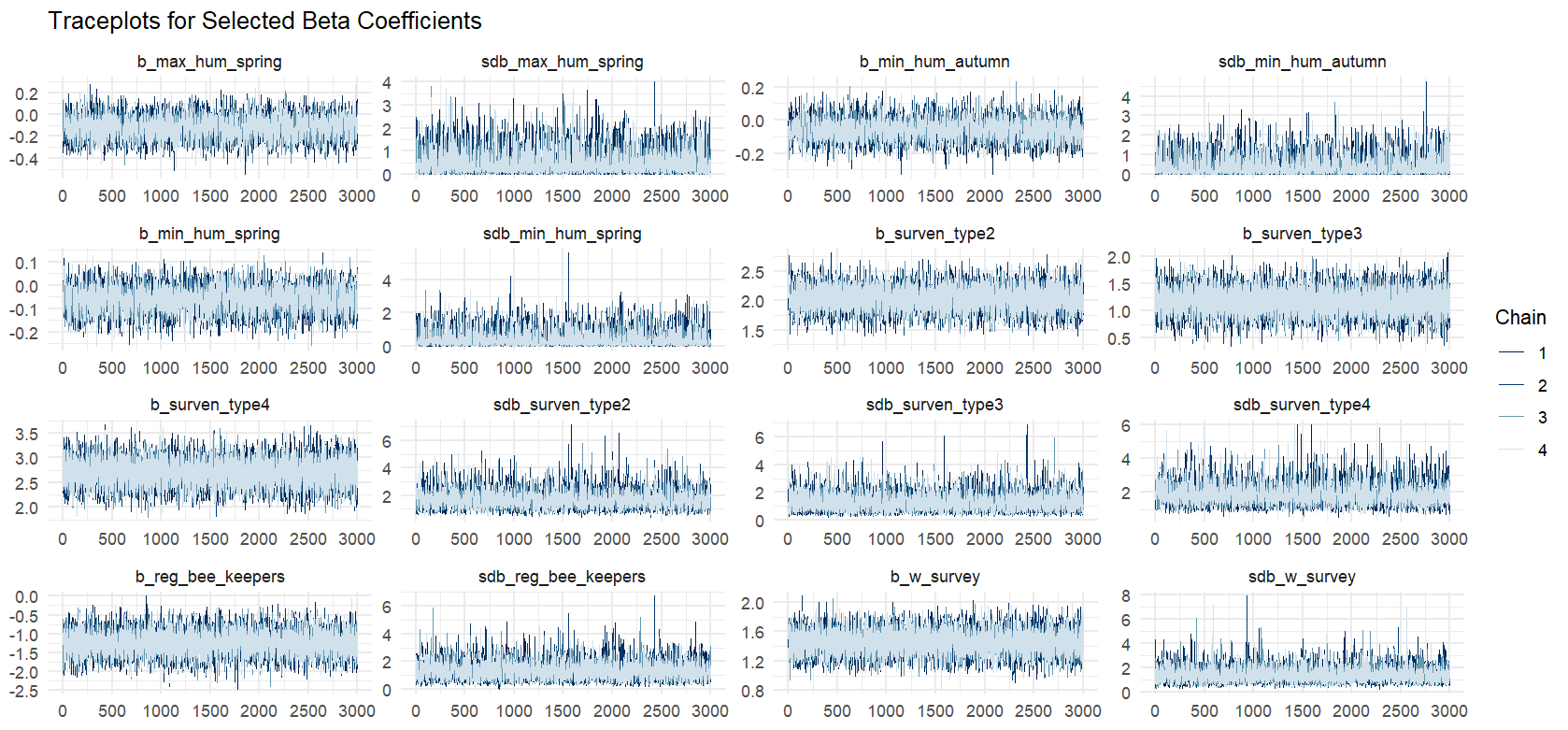


Supplementary file 1: Trace plots showing convergence diagnostics for **Model 2**


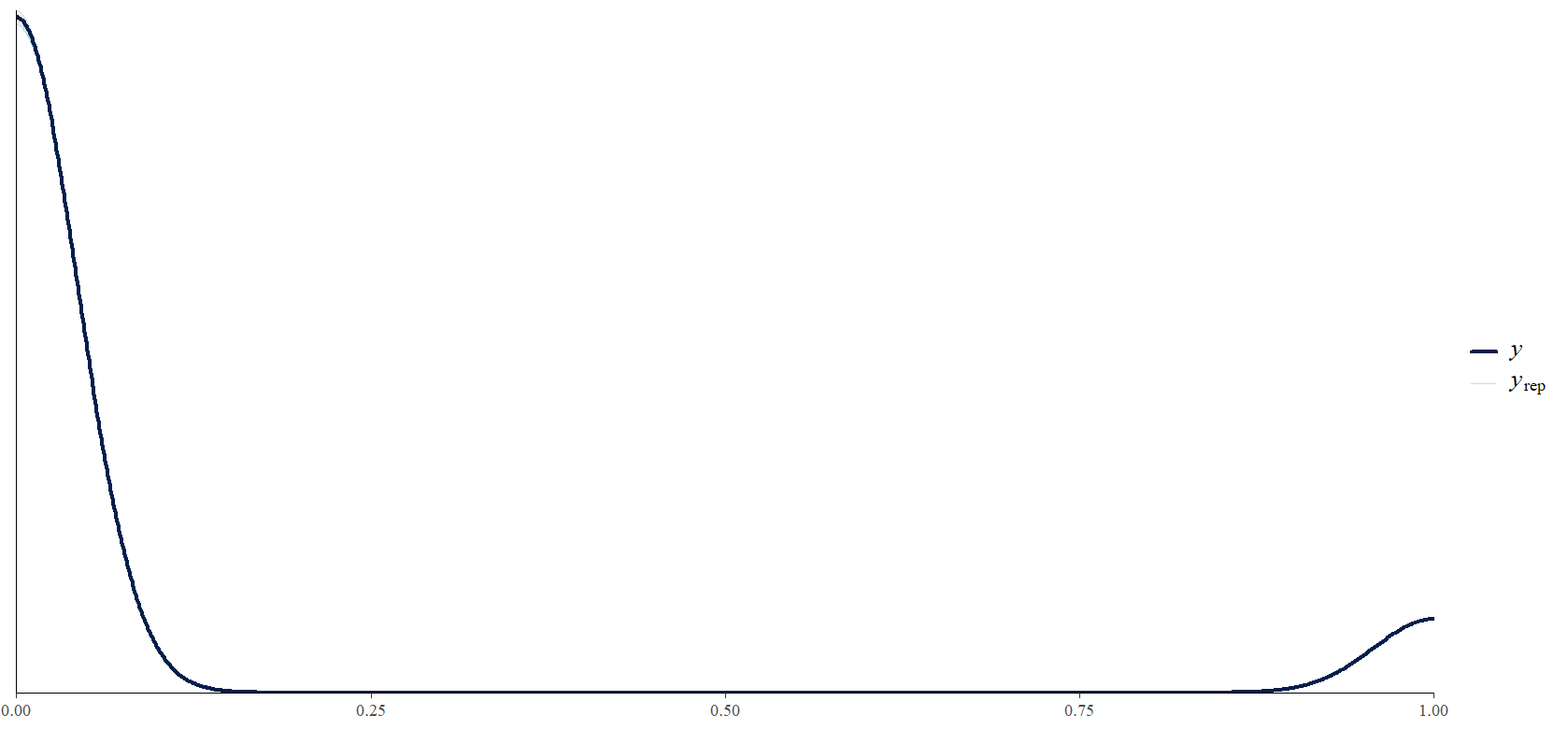


Supplementary file 1: Posterior predictive check revealed that the model (y rep) adequately reflected the variability in the observed data (y), suggesting a close agreement between the simulated and observed data further supports that the model fits well.
